# 1989- “Beef is safe.” 1996- “Not.”

**DOI:** 10.1100/tsw.2000.11

**Published:** 2000-11-03

## Abstract

A sharply critical government report on the administration of mad-cow disease research leads the news from Nature this week. Science also covers the story, but they lead with librarian's protests over a proposed merger of two science publishing giants.

Bovine spongiform encephalopathy (BSE), better known as mad-cow disease, first emerged in the late 1980s but a report released in 1989 declared that BSE posed a "remote" danger to humans, reports *Nature* in its lead story. It wasn't until 1996 that the government acknowledged that mad-cow disease had killed several people (the official BSE death count stands at 81 people, 176,000 cattle). During the delay, reports *Science*, the cannibalistic practice of feeding cattle with the remains of dead cattle, known as protein recycling, was spreading BSE "like a chain letter."

But a report released this week found that scientists had strongly suspected as early as 1992 (and proved in 1994) that BSE was being transmitted to cats, raising bright red flags that BSE could be more dangerous to people than previously thought. *Nature* reports that the mounting evidence was reviewed by the SpongiformAdvisory Committee (SEAC) in 1990 and they judged the risk to human health to be remote because potentially infective tissues from the cattle's nervous system were supposedly being removed from the human food chain. What the SEAC didn't know is that policy-makers and politicians remained convinced that BSE posed no threat to humans; therefore, they were not enforcing the regulations. *Science* reports, "Those at the 'coal face' were getting the message that there was little risk of BSE spreading to people," says Ferguson-Smith. "So they were thinking, 'What does it matter if we chuck a bit from the carcass into the wrong bin and it is processed into human food.' " Now we know. Encephalopathy

*Nature* followed up with two related stories. First, there is some evidence that beef tissue was used to manufacture oral vaccines that may in turn pass the disease to children. Second, *Nature* reports that turf battles may have delayed progress on mad-cow disease.

*Science* saved the BSE report for its second story, and lead with news that librarians are protesting European publisher Reed Elsevier's proposed $4.5 billion purchase of the American company Harcourt General. The new global company would own more than 1500 science journals, including a substantial fraction of all biomedical journals, reports *Science*. Reed Elsevier makes the familiar argument that scientists will benefit from improved efficiency. Librarians disagree. They claim that a decade of science publishing consolidation has allowed commercial publishers to increase subscription prices far faster than the rate of inflation, prompting research libraries to trim subscriptions by 6% while spending 170% more on titles. Although Reed Elsevier may be forced to sell a few overlapping titles, government regulators are expected to approve the deal. *Nature* did not cover this story.
